# Fluorescence *In Situ* Hybridization–Based Karyotyping of Soybean Translocation Lines

**DOI:** 10.1534/g3.111.000034

**Published:** 2011-07-01

**Authors:** Seth D. Findley, Allison L. Pappas, Yaya Cui, James A. Birchler, Reid G. Palmer, Gary Stacey

**Affiliations:** * National Center for Soybean Biotechnology, Division of Plant Sciences, University of Missouri, Columbia, Missouri 65211; † USDA-ARS, Iowa State University, Ames, Iowa 50011; ‡ Division of Biological Sciences, University of Missouri, Columbia, Missouri 65211

**Keywords:** soybean, chromosome translocation, FISH, karyotype, cytogenetics

## Abstract

Soybean (*Glycine max* [L.] Merr.) is a major crop species and, therefore, a major target of genomic and genetic research. However, in contrast to other plant species, relatively few chromosomal aberrations have been identified and characterized in soybean. This is due in part to the difficulty of cytogenetic analysis of its small, morphologically homogeneous chromosomes. The recent development of a fluorescence *in situ* hybridization –based karyotyping system for soybean has enabled our characterization of most of the chromosomal translocation lines identified to date. Utilizing genetic data from existing translocation studies in soybean, we identified the chromosomes and approximate breakpoints involved in five translocation lines.

Domesticated soybean (*Glycine max* [L.] Merr.) is a major crop species and the target of substantial investment of resources toward development of genetic and molecular maps, including the recently completed soybean genome sequencing project (Schmutz *et al.* 2010). However, the study of both classical and molecular cytogenetics of soybean has been relatively difficult and thus slower to develop. Very few large-scale structural aberrations have been characterized in soybean chromosomes (reviewed by Chung and Singh 2008): a few chromosomal inversion lines have been identified (reviewed by Palmer *et al.* 2000), and only seven accessions are known to involve the exchange of fragments between nonhomologous chromosomes (Palmer and Kilen 1987; Sellner, 1990; Mahama *et al.* 1999). The difficulty in characterizing even large-scale chromosomal aberrations in soybean can be attributed to their relatively large number (2n = 40; Veatch 1934), small size (∼2 µm), and morphological homogeneity (Ahmad *et al.* 1984; Singh and Hymowitz 1988). The inability to directly distinguish between different soybean chromosomes has meant that aberrations had to be characterized primarily by genetics, in combination with cytogenetics, to detect alterations in patterns of meiotic chromosome pairing. The best characterized reciprocal translocation line in soybean is *Glycine soja* P.I. 101404B, originally identified by Williams (Williams 1948) and genetically characterized by Palmer and Heer (1984). One breakpoint was positioned with respect to genetic markers on one of the two chromosomes involved (Sadanaga and Grindeland 1984; Mahama and Palmer 2003), chromosome 13 (Cregan *et al.* 1999; Cregan *et al.* 2001). This translocation appears to predominate in Chinese *G. soja* accessions (Palmer *et al.* 1987) and is indistinguishable from the exchange characterized in *G. soja* P.I. 464890B (Findley *et al.* 2010, discussed below).

The ability to rapidly and definitively address chromosome structure in soybean is now possible due to the development of a fluorescence *in situ* hybridization (FISH)-based karyotyping system (Findley *et al.* 2010), which utilizes genetically anchored bacterial artificial chromosomes (BACs; Schmutz *et al.* 2010) and variation in genomic repeats to identify each soybean chromosome. Using these tools, the translocation in *G. soja* P.I. 464890B was determined to involve the reciprocal exchange of a (>17.9 Mb) segment of chromosome 13 with a ∼4.2 Mb segment of chromosome 11 (Findley *et al.* 2010). That study was the first detailed structural analysis of any soybean chromosome rearrangement in which both the chromosomes involved and breakpoints were defined.

Fortunately, the inability to directly identify translocation chromosomes has not precluded determining whether a given soybean accession contains them, because the presence of rearranged chromosomes can be detected through their interaction with their nonrearranged (normal) counterparts in meiosis. In soybean, such interaction has been detected directly, through chromosome pairing patterns in meiosis in heterozygous chromosome translocation plants (*e.g.*, Singh and Hymowitz 1988; Mahama *et al.* 1999), or indirectly, through analysis of pollen or ovule abortion in heterozygous chromosome translocation plants (*e.g.*, Palmer *et al.* 1987). Such heterozygous soybean plants have about 50% pollen and ovule sterility (Palmer and Heer 1984). This is typical of a large number of plants that have equally frequent alternate and adjacent chromosome segregation (Burnham 1962; Endrizzi 1974). Soybean (2n = 40) normally forms 20 bivalents in metaphase I of meiosis (Veatch 1934; Singh and Hymowitz 1988). In F1 plants heterozygous for a reciprocal translocation, in addition to 18 bivalents, interaction between the normal and rearranged chromosomes can generate a single chain, or ring of four (the “☉4” conformation) chromosomes (Mahama *et al.* 1999). These aberrant, homology-based associations are thus diagnostic in confirming suspected translocations.

The power of meiotic cytogenetic analysis also can be harnessed to determine whether a *particular* chromosome is rearranged in two *different* reciprocal translocation lines (*i.e.* whether one or both exchange chromosomes are shared between two different lines, each containing a pair of reciprocally-exchanged chromosomes). For example, if line A has a translocation between chromosomes 1 and 2, and line B has a translocation between chromosomes 2 and 3, in meiosis of transheterozygous F1 plants, chains or rings of six chromosomes (☉6 conformation) will form (Burnham 1962). The presence of a ☉6 conformation in meiosis in F1 progeny generated from two different reciprocal translocation lines was a key diagnostic criterion in the analysis several soybean translocation lines (Mahama *et al.* 1999). Through meiotic chromosome interaction studies, combined with pollen/ovule sterility analyses in translocation combinations, Mahama *et al.* (1999) determined which of the seven extant soybean translocation lines had particular translocation chromosomes in common. Percentage pollen abortion, ovule abortion, and reduction in seed set were higher in F_1_ plants from crosses among homozygous translocated lines (a greater number of interchanged chromosomes) than from crosses between homozygous translocation lines (only two nonhomologous interchanged chromosomes) (Mahama *et al.* 1999). A similar inverse relationship in *Pisum* was reported by Gottschalk (1978). Despite the power of this approach, the translocation chromosomes could not be determined in these lines because the identities of the aberrant chromosomes themselves were unknown.

FISH-based soybean karyotyping (Findley *et al.* 2010) has enabled us to capitalize on genetic interaction data to characterize the remaining uncharacterized translocation lines described in (Mahama *et al.* 1999). In the present study, we focused on the following six soybean translocation lines: Clark T/T, renamed here as translocation line-1 (TL-1), which has the translocation chromosomes from *Glycine soja* P.I. 101404B introgressed into *G. max* cv. Clark; P.I. 189866 (TL-2), a naturally occurring translocation in an accession of *Glycine gracilis* Skvortz. (Skvortzow 1927), which is a weedy form of soybean from Northeast China (Broich and Palmer 1980; Shoemaker *et al.* 1986; Chen and Nelson 2004); KS175-7-3 (TL-3), KS172-11-3 (TL-4) and KS171-31-2 (TL-5), were each identified from fast-neutron irradiated *G. max* populations; and finally, L75-2083-4 (TL-6), a spontaneous translocation identified in progeny of a cross between *G. max* cultivars (Sadanaga and Newhouse 1982; Palmer and Kilen 1987; Sellner 1990; Mahama *et al.* 1999). Our analysis of these translocation lines involved sequential unraveling of the relationships detected in the cytological analysis of meiotic chromosome structures formed in plants transheterozygous for these various chromosomes.

## Materials and Methods

### Fluorescence *in situ* hybridization

Sample preparation, FISH experiments, and image processing were performed precisely as described in Findley *et al.* (2010). The Cent-Gm repeat-based FISH cocktail is based on differential chromosome painting with a cocktail of fluorophore-tagged oligonucleotides whose sequences target four variants of Cent-Gm, the soybean centromeric repeat (Vahedian *et al.* 1995; Swaminathan *et al.* 2007; Gill *et al.*, 2009; Findley *et al.* 2010). The cocktail used in Figures 3–5 utilized the following fluorophore-conjugated oligonucleotide probes (amount per slide): CY5-CentGm-2-M (7.5 ng), fluorescein-CentGm-2-E (20 ng), Texas red-615-CentGm-1-AF (0.01 ng), fluorescein-CentGm-1-G (20 ng), and CY5-SB86-C (20 ng). A fluorescein-SB86-C oligonucleotide (10 ng) was used for FISH in Figure 2, panels D and H. Images in Figure 4, panels K-L, utilized two fluorophore-conjugated Cent-Gm oligonucleotide probes (amount per slide): fluorescein-CentGm-2-E (10 ng) plus fluorescein-CentGm-1-G (10 ng) (see Findley *et al.* 2010 for oligonucleotide sequences). For all FISH and 4’,6-diamidino-2-phenylindole (DAPI) images, chromosomes presented in pairs (*e.g.* Figure 3, panels H3 and H4) were obtained from a single image of a single chromosome spread that contained both chromosome types. Exceptions were the following images that only required single chromosome types, which were obtained individually: Figures 2D; 2H; 3C; 4, C and F; 4, D and G; 4, K1–K10; 4, L1–L10; 5E3; 5F3; 5, G3–G6; 5K3; 5L4; and 5, M5–M6. Seeds germinated for isolation of root-tip chromosomes from lines characterized in the present study were derived from plants homozygous for their respective translocations. *Glycine max* (L.) Merr. cv W82 (*G. max* W82) was used as control.

As described in (Schmutz *et al.* 2010), homeologous sequences exist for the majority of regions from which BAC probes are derived; therefore, secondary hybridization signals are also frequently detectable in FISH images (Findley *et al.* 2010). However, these secondary hybridization signals are as a rule less bright, and thus distinguishable from primary signals. The hybridization signals for BAC probes in the “tester” set developed for individual chromosome identification (Findley *et al.* 2010) are well characterized. Additional BAC probes for breakpoint mapping studies were used in combination with well-characterized BACs, thereby ensuring proper chromosome identification.

### Mapping populations

The CS mutant was found in a cross of cv. SRF350 Pike in 1976 by Carol Schoener, a graduate student with Dr. W.R. Fehr at Iowa State University at Ames, Iowa. P.I. 189866 is a plant introduction from northeastern China identified as *G. gracilis* Skvortzov. It is homozygous for a chromosome translocation (X. Delannay and R.G. Palmer, unpublished results). Fertile plants in entry A04-19 (CS mutant line) segregating fertile and sterile plants were used as female parent in manual cross-pollinations with A04-152 (PI189866). The F_1_ seed was advanced to the F_2_ generation at the University of Puerto Rico/Iowa State University soybean nursery near Isabela, Puerto Rico, as entry 4. All F_1_ plants were heterozygous for the chromosome translocation and exhibited about 50% pollen sterility and reduced seed set. The F_1_ plants were single-plant threshed. The F_2_ seed were planted at the Bruner Farm near Ames, Iowa in summer 2005. Two F_2_ families (A05-187 and A05-188) segregated for the chromosome translocation and for the CS sterile that were used for the mapping study. Mature flower buds were collected from each plant separately and placed into vials containing 70% (v/v) ethanol. The anthers were squashed and pollen dispersed in a drop of 1% (v/v) I_2_KI solution (Jensen 1962). Plants were classified as fertile, about 50% pollen sterile, or male sterile by observations of pollen grain stain intensity at 100× magnification. At maturity, F_2_ plants were single-plant threshed. The F_2:3_ progenies were grown at the Bruner Farm in 2006. Approximately 50 to 60 seeds per progeny row were tractor planted in 3-m-long rows, spaced 0.7 m apart. Progeny rows were classified for segregation of the CS sterile mutant and the semisterility of the heterozygous chromosome translocation. The genotype of each individual F_2_ plant was rationalized based upon the segregation pattern of its F_2:3_ progeny row.

### SSR analysis

Ten grams of young trifoliates were collected from each F_2_ plant and stored in individual plastic bags and freeze-dried for 48 to 72 hr. Dry leaves were transferred to 15 mL sterile propylene tubes, ground to powder using glass beads, and kept at −80°C until DNA extraction. The protocol for DNA extractions of Kato and Palmer (2004) was followed. PCR reactions were performed under one of two conditions. In one set of reactions, 30 µl reactions contained 1X Reaction Buffer (10 mM Tris-HCl (pH 9.0 at 25°C), 50 mM KCl, 0.1% Triton X-100; Promega), 0.15 mM each dNTP, 1.75 mM MgCl_2_, 1.7 ng/µl genomic DNA, 0.15 µM forward primer, 0.15 µM reverse primer, and 0.1 U Taq polymerase (Promega). DNA was amplified for 32 cycles with denaturation at 94°C for 45 sec, annealing at 47°C for 45 sec, and extension at 68°C for 45 sec. In the other set of reactions, 20 µl reactions contained 1X Thermopol Reaction Buffer (20 mM Tris-HCl, 10 mM (NH_4_)_2_SO_4_, 10 mM KCl, 2 mM MgSO_4_, 0.1% Triton X-100, pH 8.8 at 25°C; New England Biolabs), 0.20 mM each dNTP, 2.5 ng/µl genomic DNA, 0.5 µM forward primer, 0.5 µM reverse primer, and 0.025 U/µl Taq polymerase (New England Biolabs). DNA initially was denatured at 95°C for 2 min, followed by 30 cycles of denaturation at 95°C for 30 sec, annealing at 45°C for 30 sec, and extension at 68°C for 30 sec, and a final extension step at 68°C for 7 min. A PTC-100 Programmable Thermal Controller (MJ Research) was used for all reactions.

PCR amplicons were analyzed on 3%–5% High Resolution Blend agarose (Amresco) containing 0.20 µg/µl ethidium bromide in 1X TBE buffer (0.089 M Tris base, 0.089 M boric acid, and 2 mM ethylene-diaminetetraacetic acid). Gels were run at a constant 80-100 V for 1.5-4 hr and visualized using UV-transillumination. Amplicons from F2 plants were noted as having either a parental or nonparental genotype. Linkage groups (including pseudo-linkage groups) were determined using QuadMap (Durrant *et al.* 2006) and MapMaker 3.0 (Lander *et al.* 1987). MapMaker 3.0 was further used to determine marker order and distances. Graphical representations of the maps were generated by MapChart 2.1 (Voorrips 2002).

## Results

### Analysis of translocation line TL-2

TL-2 was reported as a homozygous chromosome translocation line by Delannay *et al.* (1982). A cross of cv. Minsoy (PI27890) x CS mutant was used to molecularly map the CS mutant with SSRs to Classical Genetic Linkage Group 3 (*i.e.*, CGLG3; Pappas and Palmer, unpublished results). CGLG3 corresponds to Molecular Linkage group D1a (Cregan *et al.* 1999) and the pseudomolecule for *G. max* chromosome 1 (Gm01) (Schmutz *et al.* 2010). The CS locus was previously found to be linked to the translocation breakpoint in the TL-2 line (Mahama and Palmer 2003). While this information identified the involvement of chromosome 1 in the translocation, the identity of the other chromosome had to be determined by other means. To identify SSR markers that could be used for molecular mapping, the TL-2 line and CS mutant were screened for polymorphisms at several SSR loci on each chromosome (data not shown). These results allowed for the identification of *Glycine gracilis* (Gg) linkage groups between markers on Gg01 and Gg08. PCR amplification of polymorphic SSR markers from Gg01 and Gg08 (supporting information, Table S1) was performed on genomic DNA from 174 F2 progeny.

**Figure 1:**
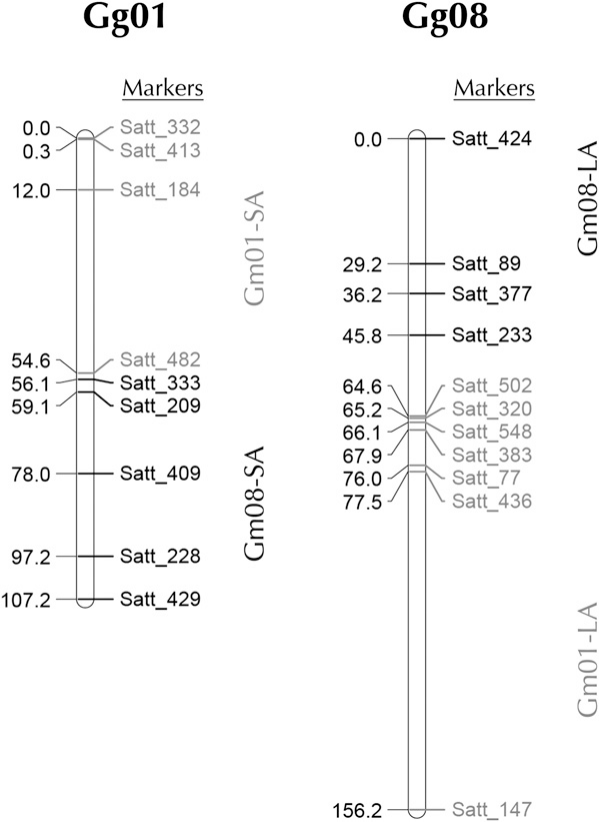
Genetic linkage maps of Gg01 and Gg08 from *G. gracilis*. SSR markers from Gg01 are in gray; SSR markers from Gg08 are in black. Several markers from the short arms of Gg01 and Gg08 are closely linked. Using information about centromere association from the FISH analysis, all short arm markers from Gg01 and Gg08 were mapped to the same chromosome (left map) as were all long arms markers (right map). Map distances are shown in centiMorgans (cM).

Several SSR markers from Gg01 and Gg08 were found to be linked. Initial analysis involved evaluating linkage groups using the program QuadMap (Durrant *et al.* 2006), which identifies linkage groups in lines with heterozygous translocations. QuadMap was expected to identify four linkage groups, each corresponding to chromosome segments on either side of the translocation breakpoints in the TL-2 line; however, QuadMap was unable to tease apart the pseudolinkage groups, including linkage of several SSR markers from the short arms of Gg01 and Gg08. The inability of QuadMap to identify the translocated segments of TL-2 prevented localization of the translocation breakpoint using molecular mapping alone, as several different maps with similar LOD scores could be generated in MapMaker. To correctly identify the region of the translocation breakpoints in Gg01 and Gg08 (Figure 1), it was necessary to use information about centromere association supplied from the FISH experiments (described below). We generated maps that show linkage of the long arm markers from chromosomes one and eight with a distance of ∼19 cM between the two closest SSRs (Figure 1, right). Perhaps most striking was the close linkage between Satt_482 and Satt_333 from the short arms of chromosome one and eight, respectively (Figure 1, left). A 1.5-cM distance between these two SSRs reflects their pericentromeric locations on the same segment.

**Figure 2:**
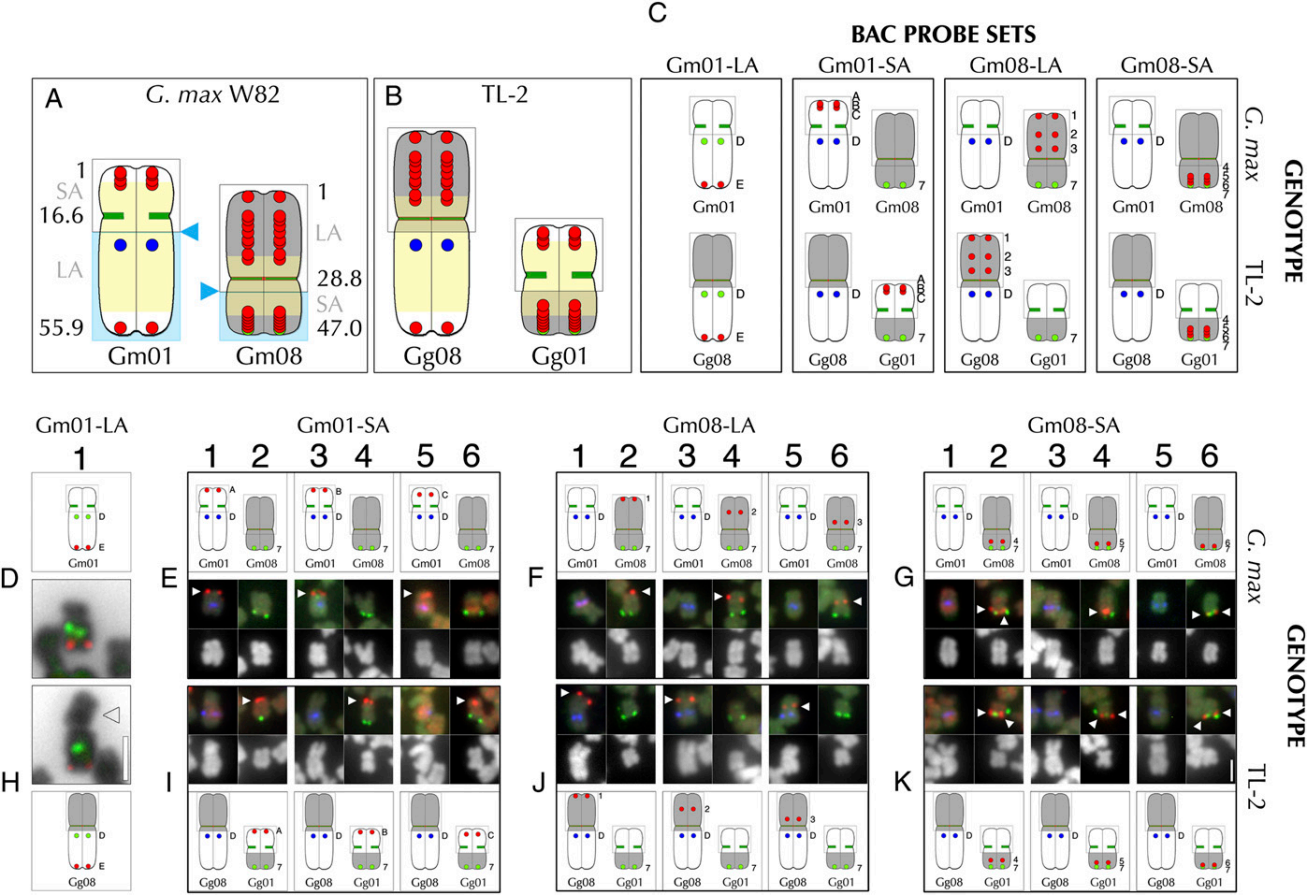
FISH-based characterization of translocation line TL-2. (A) Diagram of Gm01 and Gm08 in *G. max* W82. Numbers indicate positions (in bp or Mb) in the pseudomolecule of each chromosome. For Gm01, the short arm (“SA”) is comprised of the 5′ end (bp “1”) through the centromere region (at ∼16.6 Mb); the long arm (“LA”) extends from the centromere to the 3′ end (at 55.9 Mb in the pseudomolecule). For Gm08, the long arm (“LA”) is comprised of the 5′ end (bp “1”) through the centromere region (at ∼28.8 Mb); the short arm (“SA”) extends from the centromere to the 3′ end (at 47.0 Mb). The yellow shading depicts the approximate extent of high-repeat content sequence that defines pericentromeric chromatin. The paired red dots indicate positions of BAC probes used in mapping experiments (representative FISH images are shown; see Table S2 for complete series). The blue triangles indicate the approximate breakpoint positions for TL-2; the gray outlines represent chromosome structure unaffected by the translocation. The blue shading indicates the chromosome sections that the BACs were mapped relative to in (D–K). (B) Model of the TL-2 translocation chromosomes. Gg08 (*Glycine gracilis* chromosome 8) is comprised of sequences homologous to Gm08-LA, the Gm08 centromere, and the Gm01-LA; Gg01 is comprised of sequences homologous to Gm01-SA, the Gm01 centromere and the Gm01-SA. (C) Data summary of the probe sets used in FISH for *G. max* W82 (D–G) and TL-2 (H–K). (D1) *G. max* Gm01 hybridization with Gm01-LA markers: SB86 repeat probe (“D” in diagram, green hybridization signal in FISH panel) and the terminal Gm01-LA BAC probe, Gm01-BAC-E (“E” in diagram, red hybridization signal in FISH panel). (H1) Hybridization to the aberrantly large TL-2 chromosome with Gm01-LA markers: SB86 repeat probe (“D” in diagram, green hybridization signal in FISH panel) and the terminal Gm01-LA BAC probe, Gm01-BAC-E (“E” in diagram, red hybridization signal in FISH panel). The arrowhead indicates the aberrantly large chromosome arm associated with the Gm01-LA-homologous probes in the mutant. The DAPI signal in (D1) and (H1) is inverted to black to accentuate chromosome structure. In the *G. max* W82 BAC mapping control experiments (E–G), the upper panels are diagrams of the FISH data (middle panels), and corresponding DAPI-stained chromosomes (lower panels). In the TL-2 BAC mapping experiments (I–K), the lower panels are diagrams of the FISH data (uppermost panels), and corresponding DAPI-stained chromosomes (middle panels). In (E–G) and (I–K), three FISH probe hybridization signals are detected: the BAC probe tested (*e.g.*, Gm01-BAC-A= “A” in diagrams, with red hybridization signal, indicated by arrowhead), a Gm01-LA marker (“D” in diagrams, indicating the SB86 repeat probe, with blue hybridization signal) and a Gm08-SA marker (“7” in diagrams, indicating Gm08-BAC-7, with green hybridization signal). (E and I) The Gm01-SA probes (Gm01-BAC-A, -B, and -C) hybridized to Gm01-SA in *G. max* W82 (E1, E3, and E5, respectively), but to the arm opposite the Gm08-SA-homologous arm in TL-2 (I2, I4, and I6, respectively). (F and J) The Gm08-LA probes (Gm08-BAC-1, -2, and -3) hybridized to Gm08-LA in *G. max* W82 (F2, F4, and F6, respectively), but to the arm opposite the Gm01-LA-homologous arm in TL-2 (J1, J3, and J5, respectively). (G and K) The Gm08-SA probes (Gm08-BAC-4, -5, and -6) hybridized to Gm08-SA in *G. max* W82 (G2, G4, and G6, respectively), and also to the Gm08-SA-homologous arm in TL-2 (K2, K4, and K6, respectively). The high hybridization background of the red probes used in the experiments in (E5), (I5), (G1), and (K1) obscures the blue SB86 hybridization signal, which more evident when the blue channel is examined individually. The 2-µm scale bar in (H1) is valid for FISH and chromosome panels (D1). The 2-µm scale bar in (K6) is valid for (E–G) and (I–K).

Based on both cytology (Figure 2D1; Findley *et al.* 2010) and genome sequence assembly (Schmutz *et al.* 2010), Gm01 is a nearly acrocentric chromosome, whose short arm (Gm01-SA) and long arm (Gm01-LA) correspond to the 5′ and 3′ ends, respectively, of the corresponding pseudomolecule (see Figure 2A). In metaphase chromosome spreads of TL-2 (data not shown), a single chromosome pair appeared aberrantly large (Figure 2H1). To determine whether this chromosome was related to chromosome 1 (Gg01) in TL-2 (*Glycine gracilis*) and to investigate its structure, we used two Gm01-specific FISH probes. This first was a Gm01 pseudomolecule-derived BAC (Gm08-BAC-E; Table S2) that hybridizes to the distal end of Gm01-LA in *G. max* W82 (Figure 2D1). The second was an oligonucleotide-based probe targeting an 86-basepair genomic repeat (SB86) that uniquely hybridizes to a centromere-proximal position on Gm01-LA (Figure 2D1; Findley *et al.* 2010). In TL-2, both probes hybridized to the same arm of the aberrant chromosome, suggesting that much of the Gm01-LA-homologous segment was intact in this accession (Figure 2H1). However, the opposite chromosome arm was much longer in TL-2 in comparison to the Gm01-SA (Figure 2, D1 and H1).

Having confirmed the involvement of chromosome 1 in the TL-2 translocation, we sought to characterize the segment juxtaposed to the Gm01-LA-homologous segment in this accession by utilizing the collection of chromosome-assigned BAC probes developed for FISH-based karyotyping of *G. max* and *G. soja* (Findley *et al.* 2010). To test for a novel association of other chromosome sequences with the Gm01-LA-homologous segment in TL-2, we screened one or two BAC probes derived from each Gm chromosome by FISH. Of the 24 (of 40 possible) chromosome termini probed by these BACs, only the BAC derived from the distal end of the Gm08-LA (BAC GM_WBb0096N20; Table S2, line 31) hybridized to the chromosome containing the SB86 repeat probe target (data not shown; see Figure 2, J1, J3, and J5 for equivalent localization patterns). Thus, in TL-2, a segment homologous to Gm08-LA is associated with a chromosome segment homologous to Gm01-LA. To determine whether the translocation is reciprocal, we used positioned BAC clones (Figure 2, A and C and Table S2) to assess associations between chromosome segments homologous to Gm01-SA, Gm01-LA, Gm08-LA, and Gm08-SA in TL-2.

### Gm01-SA BACs

Having determined that much of the Gm01-LA-homologous region was intact in TL-2, we tested whether sequences corresponding to Gm01-SA are associated with the segment homologous to Gm01-LA. While all *G. max* chromosomes are characterized by large pericentromeric regions defined by a high content of repetitive DNA elements (Lin *et al.* 2005; Schmutz *et al.* 2010), Gm01 has an unusually extended high-repeat region (Figure 2A), which significantly constrained BAC probe selection. We tested eight Gm01-SA BACs (including Gm01-BAC-A through -C, Table S2; Figure 2C) in combination with two other probes: SB86 and a Gm08-SA terminal BAC (Gm08-BAC-7). In *G. max* W82, each of the Gm01-SA-derived BACs hybridized to Gm01-SA (Figure 2, E1, E3, and E5). In contrast, in TL-2, each of these probes hybridized to the arm opposite to the one hybridizing to Gm08-BAC-7 (Figure 2, I2, I4, and I6), thus indicating a *reciprocal* translocation between chromosomes 1 and 8 in TL-2 in which the Gg01-SA is associated with the Gg08-SA, and the Gg01-LA is associated with the Gg08-LA.

### Gm08-LA BACs

To determine the extent of Gm08-LA sequences associated with the Gm01-LA-homologous segment, we tested 15 Gm08-LA-derived BACs, (Figure 2, A and C and Table S2). In W82, these BACs hybridized to Gm08-LA at expected positions (Figure 2, F2, F4, and F6, diagrammed in Figure 2C and data not shown). In contrast, in TL-2, each of these probes hybridized to the chromosome arm opposite to the one hybridizing to the SB86 probe (Figure 2, J1, J3, and J5, diagrammed in Figure 2C and data not shown). Thus, in TL-2, the majority of the segment homologous to Gm08-LA is associated with the chromosome segment homologous to Gm01-LA, thereby explaining the greater overall length of the aberrant chromosome in this line.

### Gm08-SA BACs

Finally, we determined the extent of Gm08-SA homologous sequences that were associated with the Gm01-SA-related fragment in TL-2. We tested eight BACs (including Gm08-BAC-4 through -7, Figure 2C, and Table S2) spanning Gm08-SA. In *G. max* W82, each of these BACs hybridized to as expected (Figure 2, G2, G4, and G6 and data not shown). Similarly, each Gm08-SA probe hybridized to the chromosome arm targeted by the Gm08-SA-terminal probe (Gm08-BAC-7) in TL-2 (Figure 2, K2, K4, and K6 and data not shown). Therefore, most Gm08-SA-homologous sequences are associated with the tested Gm01-SA-homologous sequences in TL-2.

These experiments indicate a reciprocal translocation in TL-2 between chromosome 1 and chromosome 8 in which a segment homologous to Gm01-SA is associated with a segment homologous to Gm08-SA; whereas a segment homologous to Gm08-LA is associated with a segment homologous to Gm01-LA, resulting in the two chromosome forms shown in Figure 1 and 2B. More precise definition of the translocation breakpoints in chromosomes 1 and 8 was not possible using BAC clones, due to the high repeat content of the pericentromeric regions. However, we could identify translocation chromosomes based on their centromere labeling, because chromosomes in numerous *G. soja* and *G. max* lines share similar coloration/intensity of Cent-Gm cocktail hybridization (Findley *et al.* 2010 and unpublished observations). Using this approach (data not shown), we found that the Gm01-SA-BAC signals to be associated with a chromosome whose centromere color (aqua) resembled Gm01, suggesting that one breakpoint of the translocation (relative to *G. max*) occurred on the Gm01-LA (Figure 2A), resulting in the chromosome that we designate Gg01 (*Glycine gracilis* chromosome 1). However, the centromere color of the second translocation chromosome was not similar to either Gm08 or Gm01, suggesting that the centromere repeats of Gg08 are divergent. Molecular mapping of SSR markers supports breakpoints near the centromeres of both Gg01 and Gg08, with the close linkage of Satt482 (from chromosome 1) and Satt333 (from chromosome 8) being particularly striking (∼1 cM apart; Figure 1).

### Analysis of translocation line TL-4

**Figure 3:**
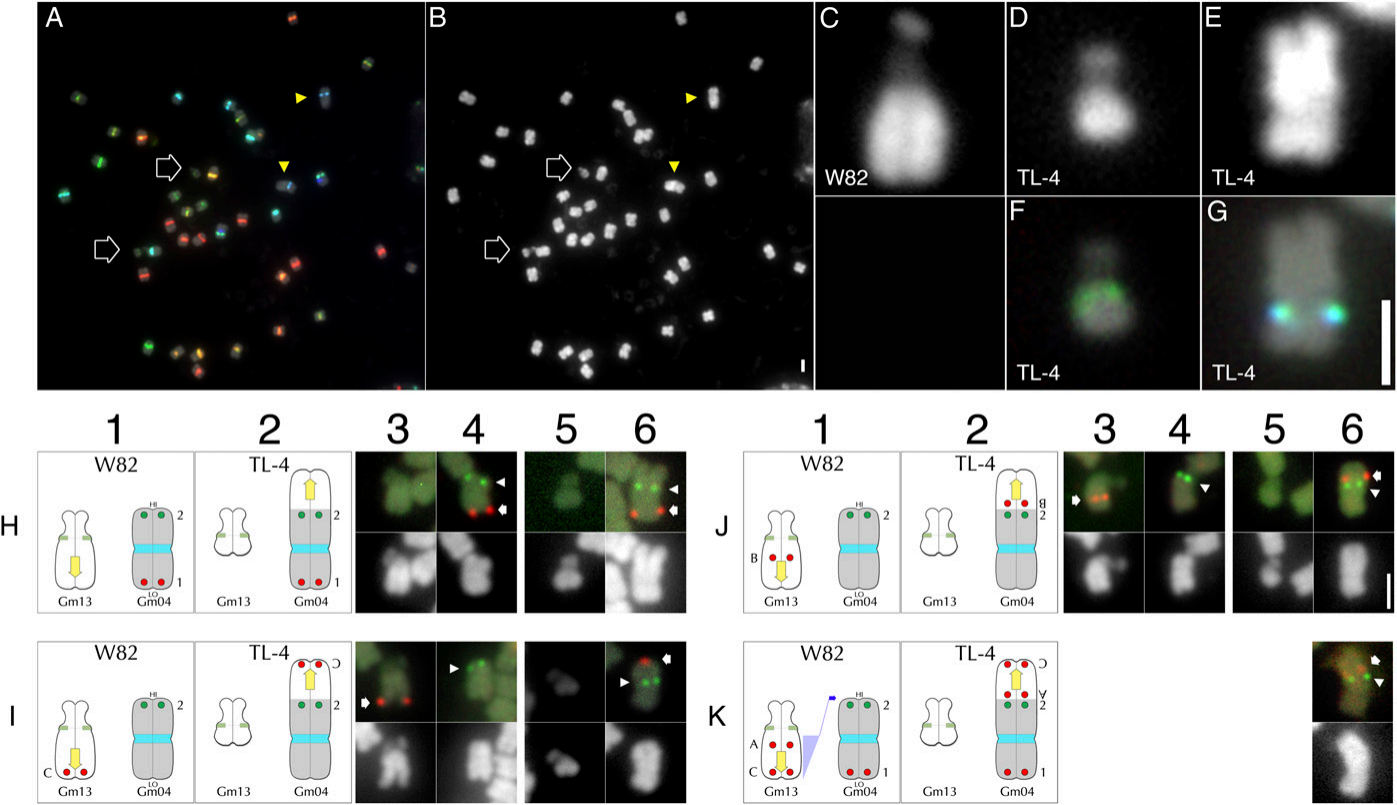
FISH-based characterization of translocation line TL-4. (A) Cent-Gm–based FISH karyotyping of a TL-4 chromosome spread; (B) DAPI channel of the chromosomes in (A). The two outline-arrows in (A and B) indicate the aberrantly small chromosome pair; the two arrowheads indicate the aberrantly large chromosome pair. (C–E) Enlarged images of DAPI-stained chromosomes: (C) a normal Gm13 in *G. max* W82, (D) the aberrantly small TL-4 chromosome, (E) the aberrantly large TL-4 chromosome. (F) and (G) show Cent-Gm–based FISH hybridization of the chromosomes in (D) and (E), respectively. (H–K) FISH utilizing pseudomolecule-derived BAC probes to map the TL-4 translocation. The *G. max* W82 chromosome diagrams in each column 1 panel correspond to the *G. max* W82 FISH images in columns 3 and 4; the TL-4 chromosome diagrams in each column 2 panel correspond to the TL-4 FISH images in columns 5 and 6. In columns 3–6, FISH hybridization panels are shown above their corresponding DAPI-stained counterparts. (Row H) In *G. max* W82 (H1), Gm04-BAC-1 (labeled “1” in column 1) hybridized to one end of Gm04 (red hybridization signal in H4, indicated by arrow); Gm04-BAC-2 (labeled “2” in column 1) hybridized to the opposite end of Gm04 (green hybridization signal in H4, indicated by arrowhead). In TL-4 (H2), Gm04-BAC-1 (labeled “1” in column 2) hybridized to the short arm of the large chromosome (red hybridization signal in H4, indicated by arrow); Gm04-BAC-2 (labeled “2” in column 2) hybridized internally, within the longer arm of this chromosome (green hybridization signal in H6, indicated by arrowhead). (Row I) In *G. max* W82 (I1), Gm13-BAC-C (labeled “C” in column 1) hybridized to the distal long arm of Gm13 (red hybridization signal in I3, indicated by arrow); whereas Gm04-BAC-2 (labeled “2” in column 1) hybridized to the 3′ end of Gm04 (green hybridization signal in I4, indicated by arrowhead). In TL-4 (I2), Gm13-BAC-C (labeled “C” in column 2) hybridized to a distal position on the longer arm of the large chromosome (red hybridization signal in I6, indicated by arrow); Gm04-BAC-2 (labeled “2” in column 2) hybridized internally, to the long arm of the same chromosome (green hybridization signal in I6, indicated by arrowhead). (Row J) In *G. max* W82 (J1), Gm13-BAC-B (labeled “B” in column 1) hybridized to a centromere-proximal position on the long arm of Gm13 (red hybridization signal in J3, indicated by arrow); Gm04-BAC-2 (labeled “2” in column 1) hybridized to the 3′ end of Gm04 (green hybridization signal in J4, indicated by arrowhead). In TL-4 (J2), Gm13-BAC-B (labeled “B” in column 2) hybridized internally within longer arm of the large chromosome (red hybridization signal in J6, indicated by arrow), close to the position of Gm04-BAC-2 (labeled “2” in column 2) on the long arm of the same chromosome (green hybridization signal in J6, indicated by arrowhead). (Row K) Translocation model for TL-4. (K1) A segment of Gm13 containing at least those sequences corresponding to Gm13-BAC-A through Gm13-BAC-C translocated (blue shading with arrow) to the 3′ end of Gm04, resulting in the TL-4 chromosome forms shown in (K2). (K3) Additional TL-4 FISH image with the most centromere-proximal Gm13 probe, Gm13-BAC-A (labeled “A” in column 2), which hybridized internally within longer arm of the large chromosome (red hybridization signal in K6, indicated by arrow), close to the position of Gm04-BAC-2 (labeled “2” in column 2) on the long arm of the same chromosome (green hybridization signal in K6, indicated by arrowhead). In columns H1–K1, the diagram for Gm04 is inverted so that the 5′ end of the chromosome (labeled “LO”) is on the bottom, whereas the 3′ end (labeled “HI”) is on the top. Unless otherwise indicated in this way in subsequent figures, the top of a chromosome corresponds to the 5′ end of the corresponding pseudomolecule. The 2-µm scale bar in (B) is also valid for (A). The 2-µm scale bar in (G) is valid for (C–G). The 2-µm scale bar in (J6) is valid for (H–K).

TL-4 formed the ☉6 conformation in translocation heterozygotes with TL-1; because TL-1 involves a translocation between Gm11 and Gm13 (Findley *et al.* 2010), it seemed likely that TL-4 would involve a translocation in one of these two chromosomes. Our strategy for characterizing this and other translocations involved three steps: first, assessment of overall chromosome morphology by examining the mutant karyotype; second, identification of candidate translocation chromosomes using the Cent-Gm cocktail in combination with chromosome-specific BAC probes; and finally, translocation chromosome verification and breakpoint estimation using positioned BAC probes (Schmutz *et al.* 2010). TL-4 chromosome spreads (Figure 3B) revealed two aberrant chromosome pairs: one that was much longer than typical soybean chromosomes (Figure 3B, arrowheads; enlarged chromosome in panel E), and a second, much shorter satellite chromosome (Figure 3B, arrows; enlarged chromosome in panel D). Cent-Gm cocktail hybridization (Figure 3A, arrowheads; enlarged chromosome in panel F) indicated that the shorter chromosome was likely a truncated form of Gm13; whereas the blue-green color of the larger chromosome centromere (Figure 3A, arrows; enlarged in panel G) suggested that it might be a modified form of Gm02, Gm04, Gm10, or Gm17. FISH with paired, chromosome-specific, terminal-position BAC probes for Gm02, Gm10, and Gm17 labeled opposite termini of single chromosome pairs with more typical morphology, indicating that these chromosomes were intact in TL-4 (data not shown). In contrast, the Gm04 terminal BAC probes (Table S2 and Figure 3H4), both labeled the aberrantly large TL-4 chromosome (Figure 3H6). The signal for the 5′ Gm04 BAC (labeled “1” in Figure 3, H1, H2, and K1) labeled near a chromosome terminus in TL-4 (Figure 3H6, red signal, indicated by arrow), as it did in *G. max* W82 (Figure 3H4, red signal, indicated by arrow), indicating that the 5′ end of TL-4 Gm04 was intact. In contrast, the 3′ Gm04 BAC (labeled “2” in Figure 3, H1, H2, and K1) hybridized internally within the aberrantly long arm, suggesting a distal addition to an otherwise intact Gm04 in this mutant.

**Figure 4:**
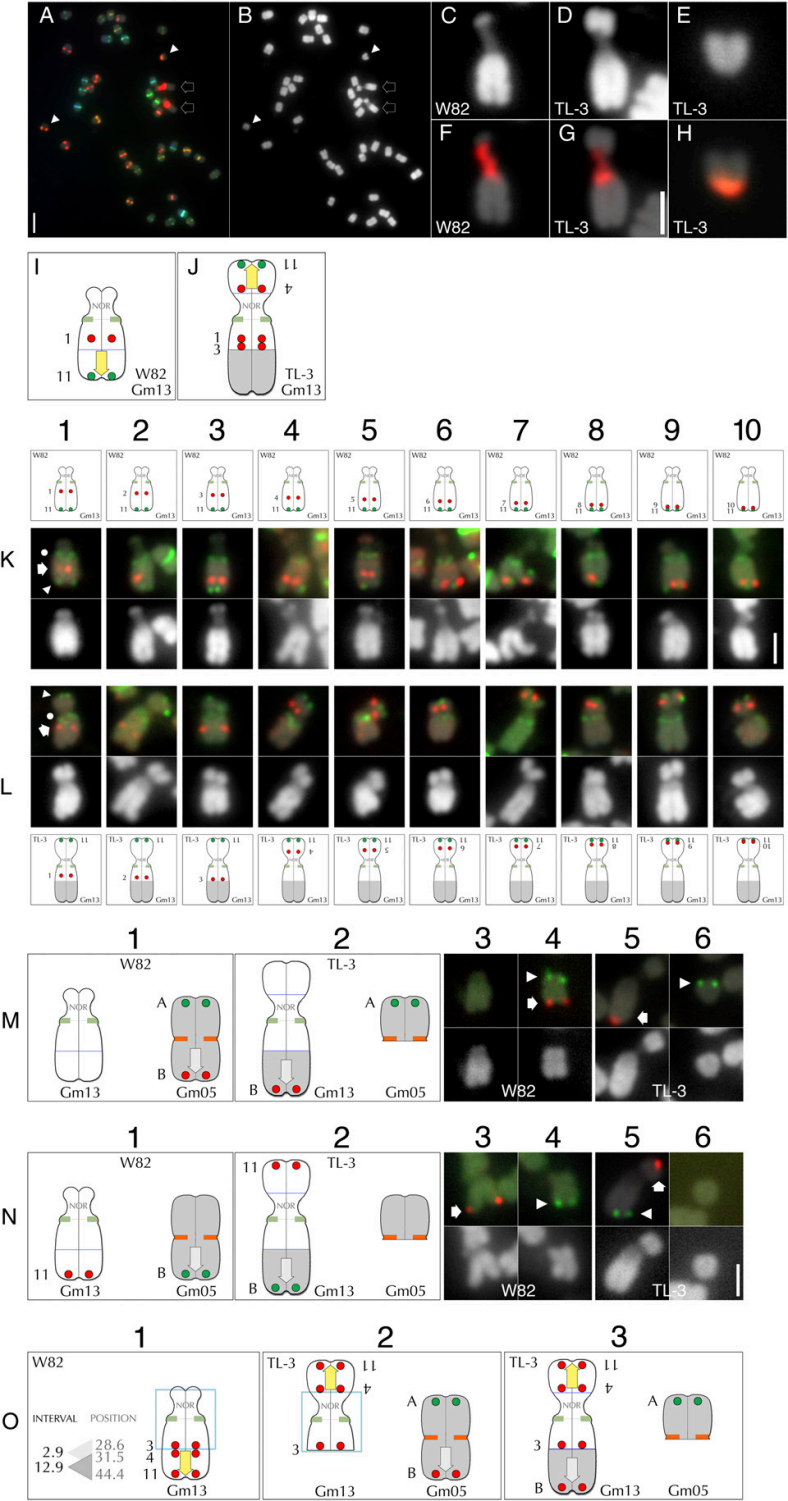
FISH-based characterization of translocation line TL-3. (A) FISH with Cent-Gm cocktail supplemented with an 18S-rDNA probe of a TL-3 chromosome spread. (B) The DAPI channel of the chromosomes in (A). The two outline-arrows in (A) and (B) indicate the aberrantly large chromosome pair that also hybridized to the rDNA probe; whereas the two arrowheads indicate the aberrantly small chromosome pair. (C–E) Enlarged images of DAPI-stained chromosomes: (C) a normal Gm13 in *G. max* W82, (D) the aberrantly large TL-3 chromosome, (E) the aberrantly small TL-3 chromosome. (F) and (G) show 18S-rDNA probe hybridization (red signal) of the chromosomes in (C) and (D), respectively; whereas the aberrantly small TL-3 chromosome in (H) is hybridized to the Cent-Gm probe cocktail. Panels (I) and (K) are diagrammatic summaries of the FISH experiments performed in (K-L) using a series of Gm13-LA BAC probes (Gm13-BAC-1 through -11; see Table S2). (K1–K10) The upper, middle, and lower panels are the chromosome diagram, FISH signal, and DAPI signal, respectively, for *G. max* W82 Gm13. The chromosome in each FISH panel has signal derived from three probes: a single tested Gm13-LA BAC (red hybridization signal, indicated in the K1 FISH panel by the arrow), plus Gm13-BAC-11 (green, chromosome-terminal hybridization signal, indicated in the K1 FISH panel by the arrowhead) and the Cent-Gm cocktail (the green, centromeric hybridization signal, indicated in the K1 FISH panel by the dot). In (K1–K10), the Gm13 BAC probes hybridized to positions spanning from Gm13-LA-proximal (K1) to Gm13-LA-terminal (K10). (L1–L10) The upper, middle, and lower panels are the FISH signal, DAPI signal, and the chromosome diagram, respectively, for TL-3 Gm13. The chromosome in each FISH panel has signal derived from three probes, as described for (K1–K10). In (L1–L10), Gm13-BAC-11 hybridized to a terminal position on the shorter arm of the aberrant Gm13 form in TL-3. (L1–L3) Only the three most centromere-proximal Gm13-BAC probes (Gm-BAC-1 through -3) hybridized near the centromere of the longer arm of the mutant Gm13. (L4–L10) Each of the remaining Gm13 BAC probes (Gm13-BAC-4 through -10) hybridized sequentially more distally, on the shorter arm of TL-3 Gm13. (M and N) FISH utilizing pseudomolecule-derived BAC probes to map the TL-3 translocation. The *G. max* W82 chromosome diagrams in each column 1 panel correspond to the *G. max* W82 FISH images in columns 3 and 4; the TL-3 chromosome diagrams in each column 2 panel correspond to the TL-3 FISH images in columns 5 and 6. In columns 3-6, FISH hybridization panels are shown above their corresponding DAPI-stained chromosomes. (Row M) In *G. max* W82 (M1), Gm05-BAC-A (labeled “A” in column 1) hybridized to one end of Gm05 (red hybridization signal in M4, indicated by arrow); Gm05-BAC-B (labeled “B” in column 1) hybridized to the opposite end of Gm05 (green hybridization signal in M4, indicated by arrowhead). In TL-3, Gm05-BAC-A (labeled “A” in column 2) hybridized to a centromere-distal position on the arm of the aberrantly small chromosome (green hybridization signal in H6, indicated by arrowhead); Gm05-BAC-B (labeled “B” in column 2) hybridized to the distal end of the longer arm of the aberrant Gm13 (red hybridization signal in (H5), indicated by arrow). (Row N) In *G. max* W82, Gm13-BAC-11 (labeled “11” in column 1) hybridized to the distal end of the Gm13-LA (red hybridization signal in (N3), indicated by arrow); Gm05-BAC-B (labeled “B” in column 1) hybridized to the 3′ end of Gm05 (green hybridization signal in (N4), indicated by arrowhead). In TL-3 (N2), Gm13-BAC-11 (labeled “11” in column 2) hybridized to a distal position on the shorter arm of the aberrant Gm13 (red hybridization signal in (N5), indicated by arrow); Gm04-BAC-B (labeled “B” in column 2) hybridized near the terminus of the longer arm of the same chromosome (green hybridization signal in (N5), indicated by arrowhead). (Row O) Model of the events that generated the chromosome forms in TL-3. (O1) Diagram of the normal Gm13. “Position” indicates the position, in Mb of the central bp of a given BAC in the Gm13 pseudomolecule (*e.g.*, Gm13-BAC-3 is at 28.6 Mb in the Gm13 pseudomolecule; see Table S2 for details); whereas “Interval” numbers represent the window in Mb between BAC probe “Position” numbers. Thus, in the first event, a chromosome break occurred in the 2.9 Mb interval between sequences corresponding to Gm13-BAC-3 and Gm13-BAC-4, thereby releasing a ∼12.9 Mb segment of the Gm13-LA. This segment then became associated with the NOR-containing satellite arm (O2, left chromosome). The yellow arrow in (O1–O3) indicates the polarity of the translocated Gm13-LA fragment; the blue box indicates the possibility that Gm13 may have experienced a pericentromeric inversion involving two breaks. Next, a fragment comprising most of 3′ end of Gm05 (O2, right chromosome) was translocated to the broken end of the Gm13-LA to generate the chromosome forms in O3. The gray arrow in (O2 and O3) indicates the polarity of the translocated Gm05 fragment. The 2-µm scale bar in (A) is also valid for (B). The 2-µm scale bar in (G) is valid for (C–H). The 2-µm scale bar in (K10) is valid for (K–L). The 2-µm scale bar in (N6) is valid for (M) and (N).

Due to the short arm length of the satellite chromosome in TL-4 (compare Figure 3D [TL-4] with panel C [*G. max* W82]), we hypothesized that a fragment of Gm13-LA might be translocated to Gm04 in this line. Indeed, Gm13-BAC-C (Table S2, line 3), which hybridizes near the terminus of Gm13-LA in *G. max* (Figure 3I3), hybridized to the terminus of the longer arm of the aberrant Gm04 in TL-4 (Figure 3I6). This result both confirmed the involvement of Gm13 in the translocation and indicated the polarity of the segment translocated; specifically, the Gm13-LA terminus now represents the terminus of Gm04-LA in TL-4. To determine how much of the Gm13-LA was translocated onto Gm04 in TL-4, we used Gm13-LA BAC probes (Findley *et al.* 2010). Gm13-BAC-B represents the most centromere-proximal BAC of a continuous series of BACs that validated the genome sequence assembly of Gm13-LA (Figure 3, J1 and J3; Findley *et al.* 2010). In TL-4, this BAC also hybridized to the longer arm of the mutant Gm04, near to the Gm04 3′ terminal probe (Figure 3, J2 and J6). Finally, although not contiguous with the other Gm13 BAC probes, Gm13-BAC-A is one of the most 5′ terminal BACs available for the Gm13 pseudomolecule; in TL-4, this probe also hybridized very near to the signal for Gm04-BAC-2, on the longer arm of the mutant Gm04 (Figure 3K6).

Altogether, our FISH mapping suggests a model (Figure 3, K1 and K2) for a translocation between Gm13 and Gm04 in which a large fragment (>17 Mb) of Gm13 has translocated to the 3′ terminus of Gm04 in TL-4. Because the most terminal Gm04 BAC tested (Gm04-BAC-2) is positioned at 44.1 Mb of the 49.2 Mb *G. max* W82 pseudomolecule, and also due to the lack of more centromere-proximal Gm13-LA probes, our analysis does not rule out the possibility of a reciprocal translocation in which a small fragment of Gm04 was transferred to the truncated TL-4 Gm13-LA.

### Analysis of translocation line TL-3

Because TL-3 formed the ☉6 conformation in translocation heterozygote combinations with either TL-1 (which involves Gm13 and Gm11; Findley *et al.* 2010) or TL-4 (which involves Gm13 and Gm04, discussed above), it seemed likely that TL-3 would have a rearranged Gm13. TL-3 had two aberrant chromosomes: one that was truncated at or near to a centromere, so that it appeared to contain only single chromosome arms (Figure 4B, arrowheads; enlarged in Figure 4E), and second that was longer than any other (Figure 4B, arrows; enlarged in Figure 4D). Furthermore, because a normal Gm13, with its characteristic NOR-containing knob (Figure 4C), was not evident in TL-3 chromosome spreads, we hypothesized that this large chromosome was an aberrant form of Gm13. We confirmed this in FISH performed using the Cent-Gm cocktail supplemented with an 18S-rDNA probe (Figure 4A). The centromere color (pale green) of the large chromosome was typical of Gm13 in other *G. max* cultivars (Figure 4A; enlargement not shown). More definitively, the central, low-intensity DAPI-stained region of the chromosome was targeted by the rDNA probe (Figure 4, D and G; compare to W82 in Figure 4, C and F), indicating that it was the NOR. Thus, the larger aberrant chromosome in TL-3 was a rearranged form of Gm13.

### Analysis of Gm13 in TL-3

To assess the chromosome structure of the TL-3 Gm13, we tested a series of BACs (see Table S2) that span most of Gm13-LA at intervals of ∼1 Mb. These probes are shown diagrammatically for *G. max* W82 in Figure 4, I and K (upper), and by FISH in Figure 4, K1–K10 (middle). The first three, most centromere-proximal probes hybridized to proximal positions along the longer arm of TL-3 Gm13 (Figure 4, L1–L3), as they do in *G. max* W82 (Figure 4, K1–K3). This preservation of colinearity of probe targets: (NOR)/(centromere)/(Gm13-BACs1-3) in TL-3 indicates a normal chromosome structure around the centromere of the aberrant Gm13 (Figure 4O, blue boxed regions). In contrast, each of the remaining Gm13-LA probes hybridized to the opposite, shorter chromosome arm of TL-3 Gm13 (Figure 4, L4–L10). The fact that Gm13-BAC-4 hybridized closest to the centromere, and Gm13-BAC-11 nearest to the terminus (Figure 4L4) of the shorter arm suggests that an otherwise intact Gm13-LA fragment was broken (between sequences corresponding to Gm13-BAC-3 and -BAC-4) and transposed to the NOR-containing chromosome arm in TL-3 (Figure 4O2). An alternative explanation to account for this novel arrangement would be an inversion (involving two chromosome breaks) of the chromosome region surrounding the Gm13 centromere. The continuity of the NOR signal in the mutant requires that one (the 5′) break of the inversion would have had to occur in the small knob region at the 5′ terminus of Gm13.

### Identification of the translocation partner in TL-3

Because the identity of the chromosome segment distal to the point at which Gm13-BAC-3 hybridized in TL-3 Gm13 (Figure 4, L3 and O2) was not resolved by the above analysis, we hypothesized that it might represent the missing arm of the aberrantly small chromosome in the mutant karyotype (Figure 4, B and E). The bright orange centromere labeling of the small chromosome (Figure 4, A and H) indicated that it could be a form of Gm05, or possibly Gm02 or Gm16, each of which is painted orange by the Cent-Gm probe cocktail in multiple *G. max* and *G. soja* accessions (Findley *et al.* 2010, and data not shown). BAC probes derived from near the 5′ end (Gm05-BAC-A) and 3′ end (Gm05-BAC-B) of the Gm05, which label opposite ends of W82 Gm05 (Figure 4M4), hybridized to two different chromosomes in TL-3. Gm05-BAC-A hybridized to the aberrantly small chromosome (Figure 4M6); whereas Gm05-BAC-B hybridized to the longer arm of the aberrant Gm13 (Figure 4M5). The fact that the Gm05-BAC-A–hybridizing chromosome arm, corresponding to the 5′ end of the Gm05 pseudomolecule, is still associated with the centromere in the mutant indicates that this chromosome was broken on the opposite arm, but close to the Gm05 centromere. Translocation of this Gm05 fragment to a rearranged Gm13 thus accounts for the longer Gm13 arm in TL-3. The orientation of the fragment in Gm13 is indicated by hybridization of the Gm05-BAC-B probe; this probe hybridizes to the distal end of the 3′ end of a normal Gm05, but hybridized to the distal end of the TL-3 Gm13-LA. This hypothesis was confirmed using Gm13-BAC-11 in combination with Gm05-BAC-B, BACs that represent 3′ positions in their respective chromosomes in *G. max* W82 (Figure 4, N3 and N4), but opposite ends of Gm13 in TL-3 (Figure 4N5). Collectively, these data are consistent with a two-step translocation model (Figure 4, O1–O3). In the first step, a distal fragment of Gm13-LA was transposed to the end of the NOR-containing Gm13-SA. In the second step, most of the 5′ arm of Gm05 was transposed to the remaining Gm13-LA, resulting in the chromosome forms shown in Figure 4O3.

### Analysis of translocation line TL-5

**Figure 5:**
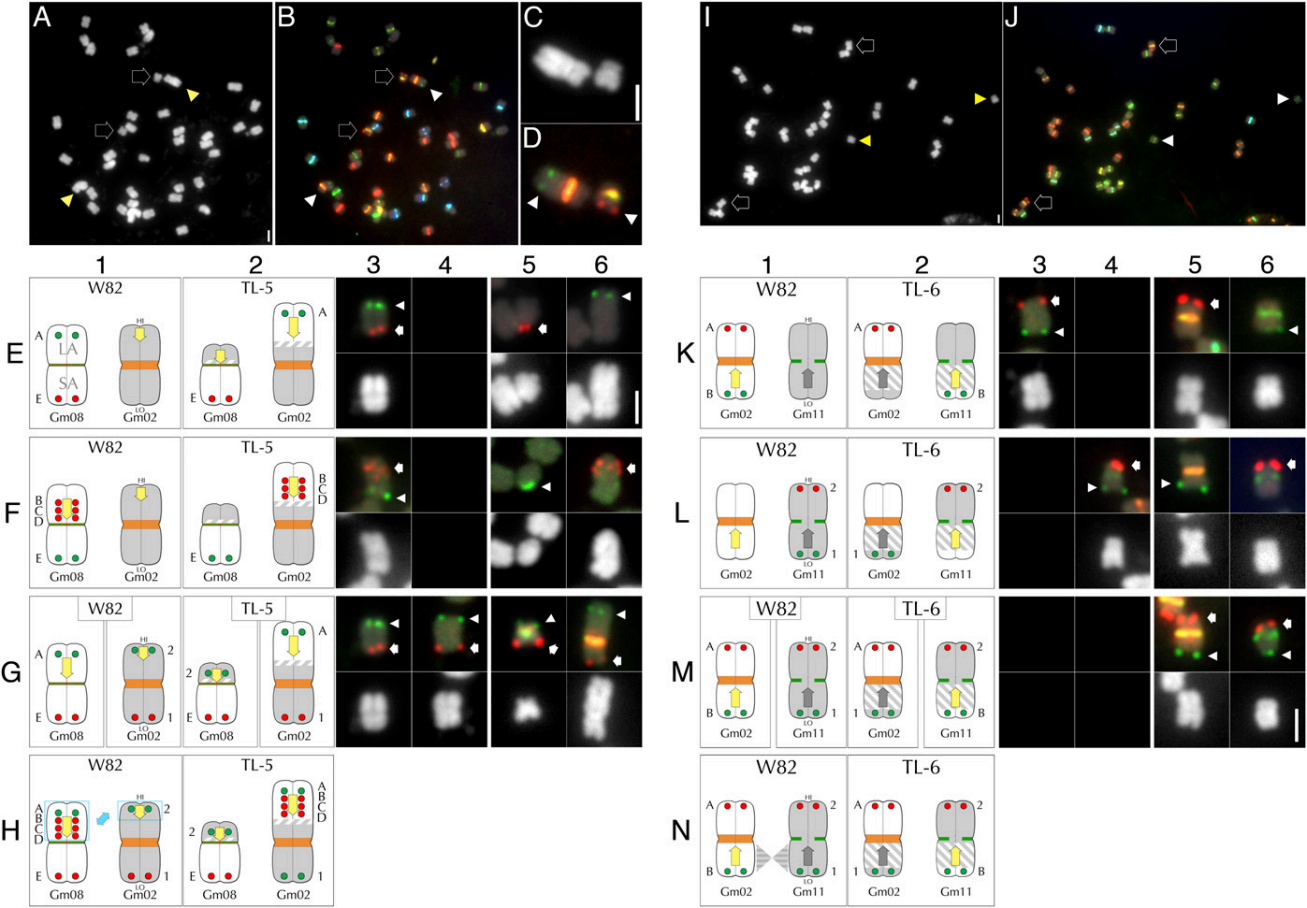
FISH-based characterization of translocation lines TL-5 and TL-6. (A) DAPI channel of a TL-5 chromosome spread. (B) Corresponding Cent-Gm–based FISH karyotyping of the chromosomes in (A). The two outline-arrows in (A) and (B) indicate the aberrantly small chromosome pair; the two arrowheads indicate the aberrantly large chromosome pair. (C) Enlarged DAPI image containing one each of the aberrant chromosome types in (A). (D) Corresponding FISH image for (C), in which the two Gm08-related BAC probes hybridized to two different chromosomes. The aberrantly large chromosome, with the bright orange centromere, contains hybridization signal (green spots) for Gm08-BAC-A; the aberrantly small chromosome, with the yellowish centromere, contains hybridization signal (red spots) for Gm08-BAC-B. (E–G) FISH utilizing pseudomolecule-derived BAC probes to map the TL-5 translocation. The *G. max* W82 chromosome diagrams in each column 1 panel correspond to the *G. max* W82 FISH images in columns 3 and 4; the TL-5 chromosome diagrams in each column 2 panel correspond to the TL-5 FISH images in columns 5 and 6. (E4) and (F4) contain no images. In columns 3–6, FISH hybridization panels are shown above the corresponding DAPI-stained chromosomes. (Row E) In *G. max* W82, Gm08-BAC-A (labeled “A” in column 1) hybridized to Gm08-LA (green hybridization signal in (E3), indicated by the arrowhead); Gm08-BAC-E (labeled “E” in column 1) hybridized to Gm08-SA (red hybridization signal in (E3), indicated by the arrow). In TL-5, the two Gm08-BAC probes hybridized to two different chromosomes. Gm08-BAC-A (labeled “A” in column 2) hybridized to the longer arm of the aberrantly large chromosome (green hybridization signal in (E6), indicated by the arrowhead); whereas Gm08-BAC-E (labeled “E” in column 2) hybridized to the longer arm of the aberrantly short TL-5 chromosome (red hybridization signal in (E5), indicated by the arrow). (Row F) In *G. max* W82, three Gm08-LA probes (Gm08-BAC-B through -D, labeled “B” through “D” in column 1) hybridized to the Gm08-LA (red hybridization signals in (F3), indicated by the arrow), and the Gm08-SA probe (Gm08-BAC-E, labeled “E” in column 1) hybridized to the Gm08-SA (green hybridization signals in (F3), indicated by the arrowhead). (F6) In TL-5, the three Gm08-LA probes hybridized to the long arm of the aberrantly large TL-5 chromosome (red hybridization signals in (F6), indicated by the arrow), and the Gm08-SA probe (Gm08-BAC-E, labeled “E” in column 2) hybridized to the longer arm of the aberrantly short TL-5 chromosome (green hybridization signals in (F5), indicated by the arrowhead). (Row G) In *G. max* W82, the Gm08-LA probe (Gm08-BAC-A, labeled “A” in column 1, left) and Gm08-SA probe (Gm08-BAC-E, labeled “E” in column 1, left) hybridized to opposite arms of Gm08. Panel (G3) is duplicated from (E3). Similarly, the Gm02-5′ probe (Gm02-BAC-1, labeled “1” in column 1, right) and Gm02-3′ probe (Gm02-BAC-2, labeled “2” in column 1, left) hybridized to opposite arms of Gm02 (E4). In contrast, in TL-5, the Gm02-3′ probe (Gm02-BAC-2, labeled “2” in column 2, left) hybridized (G5) to the chromosome arm opposite to the one with the Gm08-SA probe signal (Gm08-BAC-E, labeled “E,” in column 2, left). This centromere color of this chromosome indicates that it is a rearranged Gm08. Conversely, the Gm08-LA′ probe (Gm08-BAC-A, labeled “A” in column 2, right) hybridized (G6) to the chromosome arm opposite to the one with the Gm02-5′ probe signal (Gm02-BAC-1, labeled “1” in column 2, right). This centromere color of this chromosome indicates that it is a rearranged Gm02. (Row H) Translocation model for TL-5. (H1) A segment of Gm08-LA containing at least sequences corresponding to Gm08-BAC-A through Gm08-BAC-D has was reciprocally exchanged (blue boxes and arrow) with a segment of the 3′ end of Gm02, resulting in the TL-5 chromosome forms shown in (H2). The yellow arrows in the Gm08 diagrams indicates the polarity of the translocated fragment as determined by these experiments; the orientation of the arrows in Gm02 are conjecture. (I) DAPI channel of a TL-6 chromosome spread. (J) Corresponding Cent-Gm-based FISH karyotyping of the chromosomes in (I). The two outline-arrows in (I) and (J) indicate the mutant Gm02 pair; the two arrowheads indicate the mutant Gm11 pair; see (K5) and (K6) for enlargements. (K–M) FISH utilizing pseudomolecule-derived BAC probes to map the TL-6 translocation. The *G. max* W82 chromosome diagrams in each column 1 panel correspond to the *G. max* W82 FISH images in columns 3 and 4; the TL-6 chromosome diagrams in each column 2 panel correspond to the TL-6 FISH images in columns 5 and 6. Panels (K4), (L3), (M3), and (M4) contain no images. In columns 3–6, FISH hybridization panels are shown above the corresponding DAPI-stained chromosomes. (Row K) In *G. max* W82, Gm02-5′ BAC probe (Gm02-BAC-A, labeled “A” in column 1; red signal, indicated by arrow in K3) and Gm02-3′ BAC probe (Gm02-BAC-B, labeled “B” in column 1; green signal, indicated by arrowhead in K3) hybridized to opposite ends of Gm02. In TL-6, the two Gm02-BAC probes hybridized to two different chromosomes. Gm02-BAC-A (labeled “A” in column 2) hybridized to a chromosome with an orange centromere (red hybridization signal in (K5), indicated by the arrowhead); Gm02-BAC-B (labeled “B” in column 2) hybridized to a chromosome with a dark green centromere (green hybridization signal in (K6), indicated by the arrow). (Row L) In *G. max* W82 (L4), Gm11-LA BAC probe (Gm11-BAC-1, labeled “1” in column 1; green signal, indicated by arrowhead in L4) and Gm11-SA BAC probe (Gm11-BAC-2, labeled “2” in column 1; red signal, indicated by arrow in L4) hybridized to opposite ends of Gm11. In TL-6, the two Gm11-BAC probes hybridized to two different chromosomes. Gm11-BAC-1 (labeled “1” in column 2) hybridized to a chromosome with an orange centromere (green hybridization signal in (L5), indicated by the arrowhead); Gm11-BAC-2 (labeled “2” in column 2) hybridized to a chromosome with a dark green centromere (red hybridization signal in (L6), indicated by the arrowhead). (Row M) In TL-6, the Gm02-5′ probe (Gm02-BAC-A, labeled “A” in column 2, left) hybridized (M5) to the chromosome arm opposite to the one with the Gm11-LA probe signal (Gm11-BAC-1, labeled “1” in column 2, left). This centromere color of this chromosome indicates that it is a rearranged Gm02. Conversely, the Gm11-SA′ probe (Gm11-BAC-2, labeled “2” in column 2, right) hybridized (M6) to the chromosome arm opposite to the one with the Gm02-3′ probe signal (Gm02-BAC-B, labeled “B” in column 2, right). This centromere color of this chromosome indicates that it is a rearranged Gm11. (Row N) Translocation model for TL-6. (N1) A segment of 3′ Gm02 containing sequences corresponding to Gm02-BAC-B was reciprocally exchanged with a segment of Gm11-LA, resulting in the TL-6 chromosome forms shown in (N2). The yellow and gray arrows in all diagrams indicate the presumed, but unverified polarity. In addition, the gray hatching in all TL-6 chromosome diagrams indicates the uncertainty regarding chromosome break positions. The 2-µm scale bar in (A) is also valid for (B). The 2-µm scale bar in (C) is also valid for (D). The 2-µm scale bar in (I) is valid for (J). The 2-µm scale bar in (E6) is valid for (E–G). The 2-µm scale bar in (M6) is valid for (K–M).

TL-5 formed the ☉6 conformation and exhibited partial pollen sterility in translocation heterozygotes with TL-2, a translocation that we determined (above) involves Gm01 and Gm08. An aberration in one of these two chromosomes was thus likely in TL-5. TL-5 had two aberrant chromosomes: an aberrantly small pair (Figure 5A, arrow; enlarged in Figure 5C) and an aberrantly large pair (Figure 5A, arrowhead; enlarged in Figure 5C). FISH mapping with Gm01 terminal BACs indicated Gm01 to be intact in TL-5 (data not shown). In contrast, Gm08 terminal probes, Gm08-BAC-A (Gm08-LA, Gm08 5′ end) and Gm08-BAC-E (Gm08-SA, Gm08 3′ end), did not hybridize to a single TL-5 chromosome (compare Figure 5, E5 and E6 with Figure 5E3). In TL-5, Gm08-BAC-A hybridized near the terminus of the long arm of the aberrantly large chromosome; whereas Gm08-BAC-E hybridized to the longer arm of the aberrantly short chromosome. This indicates the involvement of Gm08 (and not Gm01) in the TL-5 translocation and also accounts for the two aberrant chromosome forms.

### Analysis of Gm08 probes in TL-5

To characterize Gm08 in TL-5 and to identify its translocation partner, we examined the mutant karyotype using the two Gm08 BACs in combination with the Cent-Gm cocktail (Figure 5B). The Gm08-BAC-E probe hybridized to a chromosome with a yellow-green labeled centromere (Figure 5D), a color typical of Gm08 in other *G. max* cultivars (Findley *et al.* 2010). This result indicated that the smaller chromosome in the TL-5 karyotype is Gm08, because the Gm08 centromere is still associated with the 5′ end of Gm08 in the mutant, and also that the breakpoint was on the opposite arm. We then determined how much of the Gm08-LA was translocated to the aberrantly large chromosome using BACs derived from Gm08-LA positions 11.0, 14.9, and 23.3 Mb. When tested individually in *G. max* W82, each of these probes hybridized only to the Gm08-LA (data not shown); when used in combination, 5-6 distinct hybridization signals are detectable on Gm08-LA (Figure 5F3). FISH in TL-5 also produced 5-6 spots, which were restricted to the long arm of the aberrantly large chromosome (Figure 5F6). Hybridization of Gm08-BAC-A near the terminus of the long arm of the large chromosome (Figure 5D) indicates that the fragment containing the 5′ end of Gm08-LA represents the chromosome terminus.

### Identification of the Gm08 translocation partner in TL-5

Because the TL-5 Gm08-SA is intact (due to contiguity between the Gm08-SA probe hybridization and the Gm08 centromere), and also that a large proportion of Gm08-LA is translocated in the mutant, we next determined the identity of the Gm08 translocation partner. The Cent-Gm cocktail painting of the aberrantly large, Gm08-LA-containing chromosome was bright orange (Figure 5D), a color typical for Gm02, or possibly Gm05 or Gm16. We first assessed the structure of TL-5 Gm02 using BAC probes Gm02-BAC-1 and Gm02-BAC-2, which are derived from 5′ and 3′ Gm02 positions, respectively (Findley *et al.* 2010, Table S2 and data not shown). In *G. max* W82, these probes hybridized to opposite ends of Gm02 (Figure 5G4). In TL-5, the signals for these BACs hybridized to two different chromosomes: the aberrantly large chromosome and the aberrantly small Gm08. Specifically, the Gm02-BAC-1 signal hybridized to the short arm of the aberrantly large chromosome (Figure 5G6); whereas Gm02-BAC-2 hybridized to the shorter arm of the mutant Gm08 (Figure 5G5). Thus, the translocation partner for Gm08 in TL-5 is Gm02, and furthermore, the translocation is reciprocal. Despite the short length of the truncated Gm08-LA in TL-5, Gm02-BAC-1, which is derived from the 3′ end of Gm02, still hybridized to the truncated Gm08 (Figure 5G5). Due to its small size, however, we did not assess the polarity of the translocated Gm02 fragment. Overall, our analysis is consistent with the translocation model shown in Figure 5, H1 and H2, in which a large fragment of Gm08-LA is reciprocally exchanged with a fragment of the 3′ end of Gm02.

### Analysis of translocation line TL-6

Because TL-6 formed the ☉6 conformation with TL-5, candidate TL-6 translocation chromosomes included Gm02 or Gm08. However, the overall karyotype of TL-6 (Figure 5I) revealed no obvious aberrations, so we relied on FISH with the Cent-Gm cocktail and BAC probes to characterize the mutant. We first tested terminal BAC clones: Gm02-BAC-A and Gm02-BAC-B, which are derived from 5′ and 3′ Gm02 positions, respectively (Figure 5K3 and Table S2). In TL-6, Gm02-BAC-A hybridized to a chromosome with a bright orange-labeled centromere (Figure 5, J and K5), which is typical for *G. max* Gm02; this also suggested that the 5′ end of Gm02 was still associated the Gm02 centromere in the mutant. In contrast, Gm02-BAC-B hybridized to a dark green centromere-containing chromosome (Figure 5K6), which might be Gm11. We therefore assessed the structure of Gm11 in TL-6 using 5′ pseudomolecule-positioned Gm11-BAC-1 in combination with 3′ pseudomolecule-positioned Gm11-BAC-2. In *G. max* W82, these two probes label opposite ends of Gm11 (Figure 5L4). In contrast, in TL-6, the two BACs hybridized to two different chromosomes: Gm11-BAC-2 was associated with the dark green centromere (Figure 5L6), but the Gm11-BAC-2 signal was associated with the aberrant Gm02 (Figure 5L5). Thus, a fragment of Gm11-LA was translocated to the 5-end of Gm02, but the Gm11-SA was still associated with the Gm11 centromere. To verify this, we simultaneously probed the mutant with Gm02- and Gm08-derived BACs (Figure 5, M5 and M6). Indeed, the 5′ Gm11-derived signal (Gm11-BAC-1) was associated with the 5′ Gm02-derived signal (Gm02-BAC-A) on a chromosome with an orange centromere (Figure 5M5). Conversely, the 3′ Gm11-derived signal (Gm11-BAC-2) was associated with 3′ Gm02-derived signal (Gm02-BAC-B) on a chromosome with dark green centromere (Figure 5M6). These data suggest a model (Figure 5, N1 and N2) for a reciprocal translocation in TL-6 between Gm02 and Gm11, in which a section of the 5′ end-associated Gm02 arm has exchanged with a section of 5′ end-associated Gm11-LA.

## Discussion

A simple but effective FISH-based karyotyping cocktail in combination with genetically anchored, pseudomolecule-derived BACs has enabled us to rapidly characterize five soybean translocations. In the process, we validated all previously detected genetic interactions between elements of a translocation interaction network (Mahama *et al.*, 1999). Critical to this effort was the prior characterization of two elements of the network, TL-1 and TL-2. At the start of our study, we knew that TL-1 involved an exchange between chromosomes 11 and 13 (Palmer and Heer 1984; Mahama *et al.* 2003; Findley *et al.* 2010); we also knew that TL-2 involved a rearrangement in chromosome 1 (Gg01; Pappas and Palmer, unpublished results). The sequential identification of individual translocation chromosomes in these and subsequent examined lines permitted us to characterize all translocation chromosomes in the network.

For TL-2, FISH-based karyotyping verified the involvement of Gg01 and identified its translocation partner, Gg08. We also determined the involvement of both chromosomes using molecular mapping of SSRs; however, identifying the chromosomal breakpoints using this method was not possible. Pseudo-linkage groups identified by MapMaker included the linkage of the short arm SSRs of both Gg01 and Gg08 (Figure 1), in support of the FISH data. While QuadMap uses an algorithm for assessing linkage groups in lines containing heterozygous translocations (Livingstone *et al.* 2000; Durrant *et al.* 2006), it was unable to identify the translocated segments in TL-2. To use molecular mapping and QuadMap to locate the translocation breakpoints, it may be necessary to use a recombinant inbred line of TL-2. Although SSR analysis has long been an important tool for constructing genetic linkage maps (Akkaya *et al.* 1992; Cregan *et al.* 1999) and assessing soybean cultivars (Diwan and Cregan 1997), its usefulness in identifying the translocation breakpoints in the TL-2 line is limited. Considering the time and money required to produce a population for mapping, screening SSR loci for polymorphisms, and assessing SSR genotypes, obtaining a plethora of alternative linkage maps identifying several different possible translocation breakpoints can be frustrating. Our FISH analysis of TL-2 circumvented the need for growing a mapping population and facilitated the unambiguous identification of chromosomal segments involved in the TL-2 translocation. The involvement of chromosomes homologous to Gm01 and Gm08 validated the observed lack of genetic interaction of TL-2 with TL-1, TL-3, and TL-4 (Mahama *et al.*, 1999), none of which involve Gm01 or Gm08, and enabled our subsequent identification of the translocation chromosomes in TL-2 interacting lines TL-5 and TL-6.

Genetic interaction of TL-5 with TL-2 (Gg01 and Gg08) permitted identification of the first TL-5 translocation chromosome (Gm08, not Gm01); the second (Gm02) was identified through Cent-Gm–based FISH karyotyping. Of the fast-neutron mutants studied, TL-5 appears to be the only one involving a simple reciprocal translocation. The chromosomes identified (Gm02 and Gm08) also validated genetic and cytological data (Mahama *et al.* 1999), which suggested that TL-5 shared a translocation chromosome (Gm08) with TL-2 (Gm08 and Gm01), but not with TL-1 (Gm13 and Gm11), TL-3 (Gm13 and Gm05) or TL-4 (Gm13 and Gm04).

For TL-4, genetic interaction with TL-1 (involving Gm11 and Gm13) permitted identification of the first translocation chromosome (Gm13, and not Gm11); the second (Gm04) was identified through Cent-Gm–based FISH karyotyping. The involvement of Gm13 in the translocation also validated the genetic interaction of TL-4 with TL-3 (below), which also involves Gm13, as well as the lack of interaction (Mahama *et al.*, 1999) with any of the other translocation lines studied (none of which involve Gm04).

The genetic interaction (Mahama *et al.*, 1999) of TL-3 with TL-1 (Gm11 and Gm13) also permitted identification of the first translocation chromosome (Gm13, not Gm11); the second chromosome (Gm05) was identified through Cent-Gm–based FISH karyotyping. This analysis validated the lack of genetic interaction of TL-3 with TL-2, TL-5, and TL-6 (Mahama *et al.*, 1999), none of which involve either Gm05 or Gm13. Because TL-3 chromosomes were derived from fast-neutron–bombarded plants, the (minimally) two chromosome breaks (one in Gm13 and one in Gm05) required for this translocation are likely to have occurred simultaneously in the same cell, followed by the action of nonhomologous end-joining repair mechanisms that generated the chromosome forms in TL-3. Of the translocations studied by (Mahama *et al.* 1999), TL-6, genetically interacted with only one other line, TL-5. Thus, characterization of TL-6 required the prior, sequential characterization of the mutants (translocation chromosomes): TL-5 (Gm08 and Gm02) and TL-2 (Gm08 and Gm01).

As demonstrated by these experiments, molecular cytogenetics represents a powerful complement to both classical genetics and genomics. Mitotic and meiotic chromosome FISH continues to be invaluable in genome sequencing efforts. FISH played important roles, for example, in the Solanaceae Genome Project, both in the direction of sequencing efforts, through BAC-based anchoring of contigs (*e.g.*, Stack *et al.* 2009), a critical step development of minimal tiling path of BACs in development of chromosome pseudomolecules, and in the detection and closure of numerous and significant gaps, in even euchromatic regions of large genomes (*e.g.*, Szinay *et al.* 2008; Peters *et al.* 2009). BAC-based FISH can also facilitate integration of molecular marker-based genetic maps with physical maps, because the technique can readily validate (or dispute) chromosome models (*e.g.*, De Lorenzi *et al.* 2010), which can be weak in domains high in genomic repeat, or in regions with a low density of molecular markers (reviewed in Szinay *et al.* 2008). The availability of soybean-based karyotyping resources, including anchored BACs (Schmutz *et al.* 2010) and a repeat-based karyotyping cocktail (Findley *et al.* 2010) should facilitate the numerous array-based comparative genomic hybridization studies currently underway in soybean. For example, in the analysis of fast-neutron mutagenesis and TILLING (targeted induced local lesions in genomes) populations, FISH can validate deletions and trace the fate (integrity and locations) of duplicated sequences. Soybean-based FISH tools, particularly BACs, due to their potential for significant cross-species hybridization, may also be useful in comparative studies (*e.g.*, Mcclean *et al.* 2010) of related *Glycine* species (*e.g.*, wild perennial Glycine species), which may not be sequenced in the near future. Numerous soybean genome resequencing efforts are underway, including those targeting the diversity (Nichols *et al.* 2007; Lee *et al.* 2008) in soybean’s wild progenitor, *Glycine soja*. However, because large-scale chromosome rearrangements may not be easily detectable by genome resequencing (*e.g.*, Kim *et al.* 2010), FISH-based cytogenetics will undoubtedly maintain its importance by its ability to rapidly assess chromosome structure in the largely uncharacterized wild soybean germplasm collections.

## Supplementary Material

Supporting Information
