# Supplementary material for: Fluorescence In Situ Hybridization–Based Karyotyping of Soybean Translocation Lines
Source: G3 (Bethesda). 2011 Jul 1;1(2):117–29. doi: 10.1534/g3.111.000034 (PMC3276125; doi:10.1534/g3.111.000034)
Supplement: Supporting Information [file supp_1_2_117__index.html]

Supporting Information 

# Fluorescence *In Situ* Hybridization–Based Karyotyping of Soybean Translocation Lines

## Supporting Information for Findley *et al.*, 2011

**Files in this Data Supplement:**

- Table S1 - Microsoft Excel, .xlsx, 12 KB
- Table S2 - Microsoft Excel, .xlsx, 60 KB
